# NaYF_4_ Microstructure, beyond Their Well-Shaped Morphology

**DOI:** 10.3390/nano9111560

**Published:** 2019-11-04

**Authors:** Godefroy Leménager, Sandrine Tusseau-Nenez, Maud Thiriet, Pierre-Eugène Coulon, Khalid Lahlil, Eric Larquet, Thierry Gacoin

**Affiliations:** 1 Laboratoire de Physique de la Matière Condensée, École Polytechnique, CNRS, Université Paris Saclay, 91128 Palaiseau, France; sandrine.tusseau-nenez@polytechnique.edu (S.T.-N.); Maud.Thiriet@polytechnique.edu (M.T.); Khalid.lahlil@polytechnique.edu (K.L.); eric.larquet@polytechnique.edu (E.L.); 2 Laboratoire des Solides Irradiés, École Polytechnique, CNRS, Université Paris Saclay, 91128 Palaiseau, France; pierre-eugene.coulon@polytechnique.edu

**Keywords:** X-ray diffraction, crystalline structure, polarized luminescence

## Abstract

Lanthanide-doped nanoparticles are widely investigated for their optical properties. However, the sensitivity of the lanthanide ions’ luminescence to the local symmetry, useful when investigating structural environments, becomes a drawback for optimized properties in the case of poorly controlled crystallinity. In this paper, we focus on β-NaYF_4_ nanorods in order to provide a detailed description of their chemical composition and microstructure. The combination of detailed XRD analysis and TEM observations show that strong variation may be observed from particles from a same batch of synthesis, but also when considering small variations of synthesis conditions. Moreover, also the nanorods observed by SEM exhibit a very nice faceted shape, they are far from being monocrystalline and present significant local deviation of crystalline symmetry and orientation. All these structural considerations, sensitively probed by polarized emission analysis, are crucial to analyze for the development of optimal systems toward the targeted applications.

## 1. Introduction

The lanthanide doped NaYF_4_ nanoparticles (NPs) are a unique class of luminescent nanoparticles that focus increasing attention due to their efficient up-conversion properties and the ability, through colloid chemistry, to nicely play on a variety of shapes [1,2,3], length [4], and doping [5]. In the last decade, these particles have been considered for a large variety of innovative applications such as biological labeling [6], nanothermometers [7,8], rheometry [9], and anti-counterfeiting [10].

In all rare-earth doped luminescent compounds, optical properties (emission efficiency, spectral shapes) are known to result from the intrinsic properties of sensitizing ions modulated by their environment within the host matrix. The effect on spectral shape is of primary importance when d orbitals are involved (Eu^2+^, Ce^3+^), but remains significant for transitions implying only f-electron states (Eu^3+^, Yb^3+^, Er^3+^). This is well documented in the case of Eu^3+^ doped compounds, this ion being considered as a very good probe of local environment, providing good indications on site symmetry through the hypersensitive ^5^*D*_0_-^7^*F*_2_ transition [11]. Much less is known and understood about structure/property relationships in the case of up-conversion compounds, which involve more complex emission scheme and energy levels [11]. NaYF_4_, which is the emblematic compound for up-conversion, is known to exist in two different polymorphs: cubic α-NaYF_4_ and hexagonal β-NaYF_4_. The impact of the host crystal on optical properties is directly evidenced by the poor emission properties of the α-NaYF_4_ as compared to the β-NaYF_4_ [12]. As revealed by a literature survey, the exact structure of the hexagonal β-NaYF_4_ phase is not so clear [13,14,15,16,17] since three slightly different crystalline structures have been reported. The list of all samples discussed in this work and the experimental conditions for their synthesis is provided on Table 1. These structures differ from their space group and small but significant variations of their cell parameters, but also different host site symmetries for the rare-earth ions (see Table 2).

**Figure 1:**
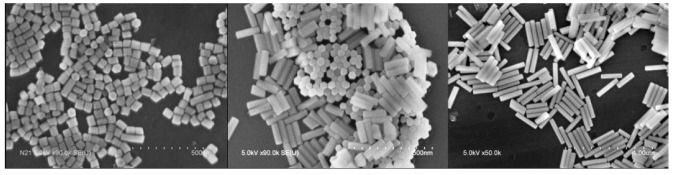
Three SEM images of NaYF_4_ NPs (from left to right NR13, NR12 and NR11).

Following our recent study on LaPO_4_:Eu nanorods [18], the initial purpose of the present work was to investigate structure/property relationship in the case of NaYF_4_:Yb-Er nanoparticles, with the objective of understanding and optimizing emission properties including emission yield, spectral shape of the emission lines and polarization properties. Using this method, polarization characterization rapidly provided evidence that such an investigation would be first limited by the fact that NaYF_4_ nanorods are apparently not monocrystalline. We found that structural investigations of NaYF_4_ nanoparticles have never been discussed in details despite the numerous works done on this kind of particles, all obtained following similar routes. We attribute this lack of interest to the very nice shape of the particles as revealed by SEM pictures (see Figure 1), which suggest an excellent quality of their structure thus hiding interest for such studies. It thus appear that polarization, usually simply explained and exploited on the basis of the intrinsic crystalline anisotropy of the hexagonal phase, is a very sensitive tool to evidence some significant deviations from ideal monocrystalline nanoparticles. All these points motivated the present study, aiming at providing new insights on the particles microstructure in order to understand and optimize optical properties that are the basis of increasing number of very exciting applications [19,20,21,22].

## 2. Experimental Section

### 2.1. Typical Synthesis of the Er^3+^/Yb^3+^/Gd^3+^-NaYF_4_ Nanorods

GdCl_3_·6H_2_O (99.99%), YCl_3_·6H_2_O (99.99%), YbCl_3_·6H_2_O (99.99%), ErCl_3_·6H_2_O (99.99%), NaOH (98%), NH_4_F (98%), NaF (98%) and oleic acid (90%) were all purchased from Sigma-Aldrich and used as starting materials without any further purification.

Synthesis where adapted from the protocols of [23,24], which consists in solvothermal precipitation of chloride lanthanide salts and NH_4_F in a water/ethanol/oleic acid mixture. Experimental conditions are shown on Table 1 for samples labeled NRXX for nanorods sample N°XX. In a typical experiment (e.g., sample NR11), 30 mmol (1.2 g) of NaOH in 5.6 mL of water were mixed with 20 mL of ethanol (EtOH) and 20 mL of oleic acid (OA) under stirring. To the resulting mixture were successively added 0.75 mmol (228 mg) of YCl_3_6H_2_O, 0.27 mmol (104.6 mg) of YCl_3_6H_2_O, 0.03 mmol (11.4 mg) of ErCl_3_6H_2_O, 0.45 mmol (167 mg) of GdCl_3_6H_2_O and 7.7 mmol (278 mg) of NH_4_F dissolved in 12 mL of water. The solution was then transferred into a 75 mL autoclave and heated at 200 °C for 2 h under stirring. In some cases, the NPs were heated for a longer time (2.5, 3 or 24 h instead of 2 h) to remove the α-NaYF_4_ NPs and also a sealed glass tube has been used to test higher pressure synthesis. After cooling down to ambient temperature, the resulting nanoparticles were precipitated by addition of 50 mL of ethanol, collected by centrifugation, washed with water and ethanol several times. They were finally dried and kept as a powder. For the optical experiments, we manipulate the nanorods (NRs) as aqueous dispersion to ease the manipulation and observations. In this case, a functionalization by ligand exchange is needed to ensure the good dispersion in water. About 20 mg of NaYF_4_ @oleic acid NPs are sonicated and centrifuged several times with 2 mL aqueous citrate solution (0.2 M), washed with EtOh and water to remove remaining oleic acid molecules, finally the nanorods are well dispersed in water.

### 2.2. Confocal Microscopy

The NPs were analyzed by a confocal microscopy system from Nikon (Nikon Eclipse Ti with confocal module C2 Si, NIKON FRANCE S.A.S., France). A nanomolar solution of citrate functionalized NPs in water is first sonicated 4 times 45 s (450 W Branson) to ensure the perfect dispersion of the NRs and then drop-casted on a glass coverslip. A Tsunami Ti-Saphire pulsed laser was focused on the NPs with a 60× oil objective. The polarized resolved spectroscopy was performed by means of a spectrometer (IsoPlane SCT320 from Princeton Instruments—Teledyne France, France) coupled to CCD (Pixis-400-BX) and a motorized polarizer.

### 2.3. TEM Observation

Transmission Electron Microscopy (TEM) experiments were performed using a field emission gun JEOL JEM-2010F microscope operating at 200 kV with a high-resolution HR polar piece (Cs = 1.0 mm, Cc = 1.4 mm, point resolution = 0.24 nm, with a dose of ≈20 electrons Å at 50,000 magnification and ≈100 electrons Å at 250,000 magnification). Chemical mapping was performed by XEDS (Cliff-Lorimer methods) using a FEI Titan Themis probe-corrected microscope operating at 200 kV and equipped with a Scanning Transmission Electron Microscopy (STEM) module at 115,000 and 225,000 magnification (probe size 1 Å) and “Super-X” detectors (beam convergence angle: 24.6 mrad).

During the ions quantification, an amorphization of the NPs may be observed. The loss of the crystalline structure can increase the ion mobility and change the doping repartition in the NP but not the average doping of a NP. For this reason, we present only global measurements on whole NPs.

### 2.4. X-ray Diffraction: ESRF

The synchrotron X-ray Powder Diffraction (XRPD) experiments were carried out at the Swiss-Norwegian Beam-Lines (BM1A station) of the European Synchrotron Radiation Facility (ESRF), France. A monochromatic beam of wavelength λ = 0.7129 Å was focused onto the sample by sagittal bending of the second crystal of a double-crystal Si(111) monochromator using additional slits of 272.26×327.34 μm^2^. XRPD data were collected in transmission geometry using a pixel-array detector (PILATUS 2M, Dectris Ltd., Switzerland). Samples were sealed in 0.4 mm diameter glass capillaries and rocked by 10° during data collection. The instrumental resolution was determined using a LaB_6_ NIST standard (Standard Reference Material 660a, cell parameter = 0.41569162 nm ± 0.00000097 nm at 22.5 °C).

The selected patterns for the identification of phases by XRPD and the crystallographic data (COD database [25]) for the line profile analysis are summarized in Table 2.

## 3. Results and Discussion

### 3.1. Confocal Microscopy and Polarized Luminescence

**Figure 2:**
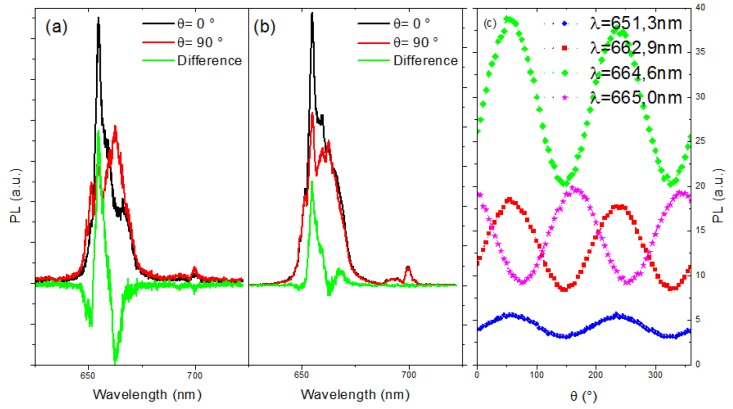
(**a**,**b**) Spectrum for two orthogonal polarizations (red and black) and the difference between them (green) for two different nanoparticles (NPs) of the same batch NR07. (**c**) PL intensity versus polarization for different wavelengths.

With our confocal set-up, the polarized spectra of our NPs deposited by drop-casting of diluted NaYF_4_ NPs in water are measured. The low concentration coupled to a sonication of the solution just prior deposition leads to dispersed NPs on the glass coverslip. Without reference, the orientation of the NP cannot be known nor the expected orientation of the polarization. We measured the spectra for a wide range of polarization angle. In these conditions, the axes of the two orthogonal electric dipoles will be given by the least brilliant and the brightest spectra. In Figure 2a we present the photoluminescence (PL) spectrum for two orthogonal polarizations (red and blue) and the difference between them in green. Moreover, by plotting the PL intensity versus the polarization angle (see Figure 2b), the typical dipole-like behavior expected from lanthanide ions appears to be in good approximation with the Malus law (see Figure 2c).

However, if we look in detail this dependence for several wavelengths, the extreme values are not obtained for the same angles as expected. Indeed, the polarization is due to the site symmetry of the lanthanide ion and the electrons can optically relax by emitting α, σ or π photons [28] with orthogonal polarization. Then a PL dependence can only present two angles for the PL optima values for all measured transitions, which is not the case: the 665.0 nm transition has a maximum shifted of 20° as compared to another transitions at 662.9 nm. This observation leads to conclude that the local symmetry orientation is not the same for all the emitters. To understand this observation, we investigated the microstructure of the particles in more details.

### 3.2. TEM Observation and Doping Homogeneity

**Figure 3:**
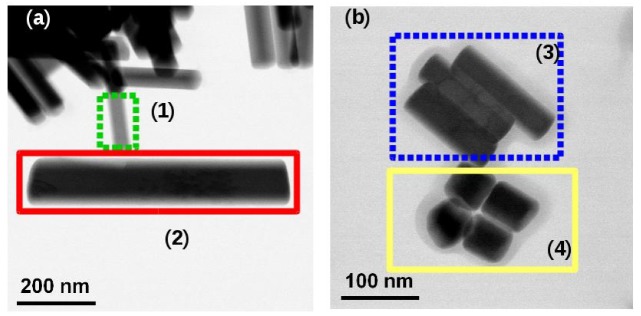
Two TEM images of NaYF_4_ NPs from the two batches presenting two NPs populations ((**a**) NR11 and (**b**) NR13).

The doping of the NaYF_4_ NPs (nature and concentration of the rare earth ions) plays an unquestionable part in the shape and structure of the NPs [3]. Several batches of NPs were synthetized with different nominal doping concentrations. The effective doping of NPs was measured by EDX analysis. The Table 3 presents the expected and effective doping of particles obtained from different syntheses and shown in Figure 3.

The three first samples were prepared under similar conditions (doping concentration, heating temperature and time). However, the effective dopings are found to be different: NRs are less rich in Y of about 20 mol%, more than 3 times more rich in Er whereas Yb and, in a minor way, Gd doping are close to the expected values. This shows the sensitivity of the synthesis to some parameters that can be associated to the volume of solvent and the type of container, that could affect for example the pressure during the synthesis (see Table 3).

TEM observations evidences two situations. On one hand, we sometime observe in the same batch two (or more) populations of NPs, each one having a relatively low size dispersion. However, the size of each population can be very different as it is illustrated in Figure 3 for two syntheses (NR11 and NR13). The EDX measurements of the delimited area give us the chemical composition as reported in the Table 3. For each population, we measure a strong difference in the gadolinium concentration (and in consequence the yttrium concentration) while erbium and ytterbium concentrations present only small variations. In Figure 3a, the NP in the area (1) delimited by the dotted green line area has a concentration of gadolinium of 32 mol% and its size is typical from this synthesis (50 × 260 nm), while the NP shown by the continuous red line (area 2) has a concentration of only 18 mol% and its size is twice the average size (110 × 650 nm) with a similar aspect ratio. Another example for the batch NR013 is presented in Figure 3b where the same behavior is observed with a complete different aspect ratio. While the experimental conditions are exactly the same, one (NR00) or two populations of particle sizes (NR11) are observed, with identical aspect ratios (about 6). At this stage, no clear parameter can explain these results but this is probably an evidence that the two kind of particles formed at different time during the synthesis, probably because the kinetic of formation of Gd rich or Gd poor particles is different.

On the other hand, the expected doping and the effective doping can differ strongly. Depending on the synthesis, the gadolinium concentration expected to be around 30%, is found to vary from 18% to 38%. Two samples share a population with the same doping while the first sample presents a different doping. The two last experiments were done with another solvent ratio compared to the first experiment resulting in a different pressure during the synthesis. These differences in pressure, solvent composition and the correlated solubility can explain the doping difference between the first and the two last syntheses.

### 3.3. Phase Identification and Doping Dependence

**Figure 4:**
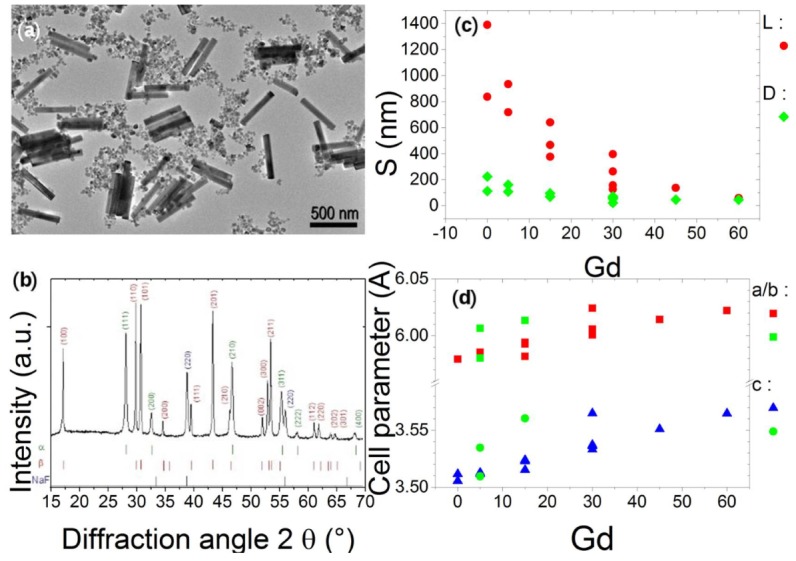
NaYF_4_ nanoparticles (**a**) Transmission Electron Microscopy (TEM) image with small cubes (α-NaYF_4_) big nanorods (β-NaYF_4_) NR01 (**b**) typical X-ray pattern of these particles (**c**) TEM NPs sizes S (L = lenght, D = diameter) and (**d**) cell parameter dependences vs Gd nominal doping with blue triangles and red squares for the main set of cell parameters of each batch and green squares and green circles for the second set of cell parameters.

The mixture of the α-NaYF_4_ and β-NaYF_4_ structures (visibles in Figure 4a) can easily be studied independently since they exhibit clearly different diffraction patterns. In Figure 4b, we present one typical diffraction pattern obtained for a mixture of theses two types of phases. Not all of our syntheses result in a mixture of α-NaYF_4_ and β-NaYF_4_ nanoparticles—we focused our work on β-NaYF_4_ nanorods as this phase provides the interesting optical properties. As previously mentioned, the β-NaYF_4_ NPs can adopt three different crystalline structures, with space groups depending on the synthesis parameters known to be P6_3_/m, P$\bar{6}$ and P$\bar{6}$2m (see Figure 4) [26]. The main difference between the powder diffraction patterns lies in an extra peak from the (001) planes for the P$\bar{6}$ and P$\bar{6}$2m compared with the P6_3_/m patterns at 2θ = 11.70° in our experimental conditions. The intensity of this peak is very low, estimated around 2% of the maximum diffraction peak intensity and below 0.1% respectively for P$\bar{6}$ and P$\bar{6}$2m. This small difference is usually not visible in XRPD done with a usual laboratory diffractometer and was at the origin of long discussions in the literature [13,14]. The P$\bar{6}$ and P$\bar{6}$2m can only be distinguished by a quantitative analysis of the peak intensities. A synchrotron facility is required to properly determine the symmetry of NaYF_4_ NPs.

For the β-NaYF_4_ NRs, among the thirteen studied samples, only one of them presents a detectable specific peak of the P$\bar{6}$ or P$\bar{6}$2m structure. Furthermore, the intensity of this peak is very low (0.1% of the maximum intensity) indicating that only a negligible portion or region of the particles was obtained with this structure. For these reasons, only the P6_3_/m structure was considered in the following for our NPs.

After the phase identification, the Fullprof software [29,30] was used in profile matching mode with a constant scale factor in order to perform a microstructural analysis. The XRPD patterns were fitted with a Thompson-Cox-Hastings function [31] (a modified pseudo-Voigt function) to extract, for each phase (α and/or β-NaYF_4_), the cell parameters and the width and breadth (full width at half maximum FWHM and integral breadth IB) of the Bragg peaks. Our study focuses on profile fitting and diffraction line broadening analysis. As said previously, the particles are doped with a constant nominal concentration of ytterbium (18 mol%) and erbium (2%) and we only changed the gadolinium concentration (from 0% to 60%). On the contrary to EDX focusing on single or few NPs, XRPD gives a global and averaged analysis of the sample.

Figure 4d presents the evolution of the cell parameters with the nominal gadolinium content of the β-NaYF_4_ phase. In some samples (NR03, NR 04 and NR06), two β-NaYF_4_ phases are identified, a main peak is observed at the expected position and a second one slightly shifted (shoulder or separated peak, associated to other set of cell parameters shown in Figure 3d with a green star or circle). We may infer that these two phases correspond to the two populations of particles that were evidenced by TEM imaging and TEM analysis (see Figure 3). Moreover, both sets present in parallel a linear dependence with the amount of gadolinium (even with the poor statistic of the second group). The second set of cell parameters cannot be explained by a population with a higher gadolinium concentration as observed previously. Yet, the linear dependency is expected due to the higher ionic radius of the gadolinium compared to yttrium.

### 3.4. Microstructural Analysis

For all the 13 studied samples, all XRPD patterns exhibit peaks broader than the instrumental resolution (typically the FWHM for the samples was above 0.050° 2θ to be compared with 0.029° 2θ for the LaB_6_ standard pattern). This broadening is known to be due to size effect and/or microconstrain in the system. From our previous fit, the shape factors (ϕ) can be defined by $\phi =IB_{vo}/FWHM_{vo}$ with $IB_{vo}$ the observed integral breath (IB) and the observed FWHM. The Lorentzian and Gaussian limits for the shape factors are respectively ϕ=2/π≈0.6366 and $\phi =2{\left(\frac{log_{e}\left(2\right)}{pi}\right)}^{1/2}\approx 0.93949$ [32]. Shape factor with ϕ < 0.6366 and ϕ > 0.9394 can be referred as super Lorentzian and super Gaussian respectively [32]. Otherwise, it implies that the profile is a convolution of both shapes and justifies the use of a pseudo-Voigt.

It is well admitted now that, for small geometrical effects of a single line instrument, the instrumental profile is approximately Lorentzian, whereas the profile arising from lattice strain is more Gaussian [33,34]. The broadening due to defaults or small crystallite size depends respectively on their nature of the shape and the size distribution of the crystallites, and is assumed to be Lorenztian. These components can be taken as Voigtian, the Lorentzian and Gaussian parts are the limiting cases. If two or more reflections are available, size and strain effects can be determined from the variation of Lorentzian and Gaussian contributions in the IB depending on the hkl Miller indices [35].

We followed the procedure recommended by Langford [32], computing the FWHM and IB of a pseudo-Voigt profile from the broadened profile to give the shape factor, calculating the breadths of the constituent profiles corrected by the instrumental broadening and analyzing the Williamson-Hall [36] and Halder-Wagner [37,38] plots. The empirical procedure derived by de Keijser et al. [35] for calculating the Lorentzian and Gaussian components (betaL, betaG) and the integral breadth (IB) has been used in the Fullprof software.

**Figure 5:**
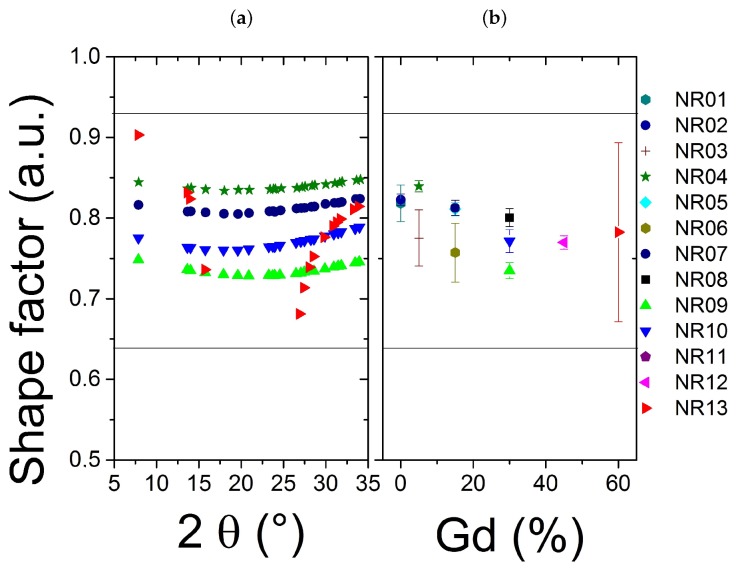
(**a**) Shape factor for each peak of four typical samples (NR04, NR07, NR09, NR10) and the most different sample (NR13) (**b**) Mean shape factor versus the expected gadolinium concentration.

The Figure 5a presents the dependence of the shape factor for four typical samples (NR 04-07-09-10) and the most different one (NR13), all the peaks show a shape factor between Gaussian and Lorentzian limits. The shape factor may not correlated with the Gd concentration (Figure 5b) To understand the origin of the broadening of our peaks, possibly due to both size and microstrain contribution, the Halder-Wagner method [37,38] is recommended with such profile parameters instead of a Williamson-Hall approach [36], as the peaks have not a pure Lorentzian shape. Note that the fit is not so dependent on the 2θ range, even if Halder-Wagner plot insists on the small 2θ values (however the most intense peaks).

**Figure 6:**
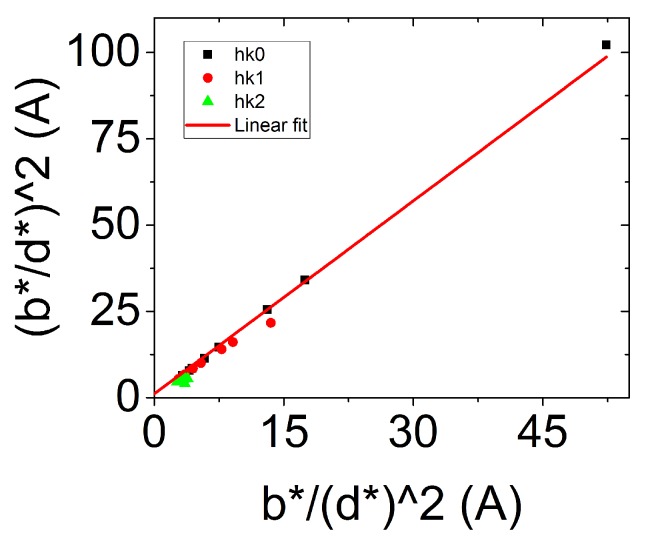
Halder-Wagner plot for a typical sample.

To distinguish between size and microstrain respective contributions, the Halder-Wagner equation is written as ${\left(IB^{*}/d^{*}\right)}^{2}=IB^{*}/{\left(d^{*}\right)}^{2}$. By plotting this equation a linear behavior is observed for all our samples (Figure 6). The intercept of the linear regression in the Halder-Wagner plot is related to the importance of microstrain in the material and, on the other hand, the slope is related to a mean apparent size (in volume) of the NPs.

In Figure 6, here for NR08, a clear linear regression is observed, with an origin close to zero. This behavior is common to all our synthesized NPs, as summarized in Table 4. Whatever the sample the microstrains are close to zero. For the rest of the study, the broadening of the Bragg peaks is then considered to be the broadening of a size effect only, and the microstrain effect can be neglected. One can also notice that the characteristic size of the NPs obtained from the Halder-Wagner plot is smaller that the one obtained by TEM imaging. In first approximation, it appears that the size obtained by XRD is close to the diameter of the NPs observed by TEM. This lead us to study more carefully the crystallinity of our NPs to understand the meaning of this characteristic size.

The previous observation was assuming an isotropic NPs shape, supposed to be spherical. Now a Scherrer-type analysis was used, where only the Lorentzian contribution is considered for each individual reflection in order to take into account eventual anisotropic broadening, to obtain the size of the crystallite, a needle-like model was applied—with a main axis along the c axis or [001] direction. Remember that by this method it is at best a mean value of the size of the coherent domains of a supposedly unimodal distribution of crystallites which is obtained for each (hkl) Bragg peak.

From this analysis, the three dimensions of the crystallites in our NPs can be extracted. All of them present a longer length along the [OOl] direction and a smaller size in the corresponding orthogonal [hk0] directions. Moreover, in most cases, the length along this direction [hk0] is slightly smaller than the apparent size observed by TEM. We summarized the crystallite sizes in the Table 5 with again the apparent size for an easier comparison.

**Figure 7:**
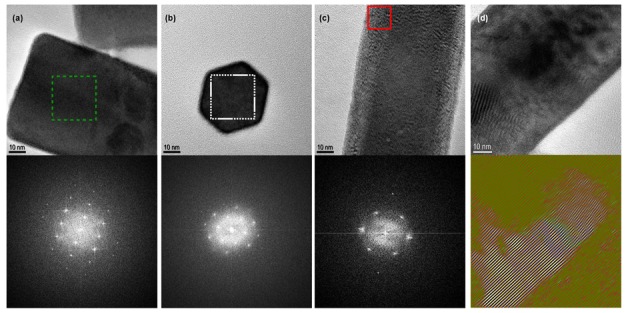
(**a**–**c**) HRTEM of NPs with below the FFT of the indicate area (**a**) NR09 with a typical FFT orthogonal to the c-axis (**b**) NR13 with a typical FFT parallel to the c-axis (**c**) NR11 with a FFT characteristic from an hexagonal phase trough the c-axis but observed orthogonally to the c-axis. (**d**) HRTEM of a NR06 with various defects below same image filtered with false color.

As previously said, the smallest length of the crystallites is associated to the directions orthogonal to c-axis. Moreover, for NPs with a diameter below 100 nm, this length is almost always slightly smaller of few nanometers than the diameter of the NPs. This fact was explained with the HRTEM observation. In Figure 7a,b, we observed an amorphous phase or a less crystallized shell around the NPs. Another observation, more surprising from our point of view, is the possibility to have a well crystallized phase in an orthogonal direction surrounding the core of the NPs. The inset of Figure 7a shows the FFT of the area delimited by the dotted green line. This FFT presents a rectangular signal characteristic from an hexagonal structure seen orthogonally of the c-axis. Even though the NPs in Figure 7c is a rod well defined, the FFT of the area delimited by the continuous red line is characteristic from an hexagonal phase through the c-axis. Note that the crystallinity of the NPs presented is not altered by the electron beam of the TEM.

This observation is important if we want to analyze the luminescence of the NPs. It is well known that lanthanide ions have a specific luminescence correlated with their local environment. For a structure like P6_3_/m, the doping ions have access to only one type of site as mentioned in Table 2. The luminescence and in particular the polarized properties will be depending of this site (here $C_{3h}^{2}$) [28]. But if the crystallinity of the NPs is not good meaning polycrystallines nanorods instead of monocrystalline and the lanthanide-site are not well aligned with a well-defined c-axis along the whole rod, the optical properties will be affected starting with a reduction of the polarization degree. Moreover, for singles NPs studies, due to statistical variation from one NP to another, it will be difficult to analyze the optical properties.

## 4. Conclusions

Many works have reported the synthesis, optical properties and application of Na(Gd)YF_4_:Yb, Er nanoparticles. The large interest for this systems comes both from their highly appealing emission properties, and the ability to synthesis particles with different size and shapes. In particular, very nice facetted nanorods can be obtained with adjustable aspect ratio playing on the particles composition such as some Gd^3^ ions in substitution to Y^3^. Nevertheless, characterizations of polarized emission from single nanorods evidence that the particles are not monocrystals, which motivated this study to get a deeper insight in the particles microstructure. This work has been done using TEM and XRD analysis on a set of 13 samples obtained under various conditions. We could show that in a given batch of synthesis, and focusing on particles with the β-NaYF_4_ structure, different populations of particles may coexist with different size and shape, but also with strong deviations from the nominal composition. XRPD analysis using the Halder-Wagner approach, combined with HRTEM point out an anisotropic polycrystalline structure of the nanorods with important local variations of the crystallites orientations. This is clearly at the origin of the polarization spectra, which is very sensitive to crystallites orientation.

As NaYF_4_ NPs are now widely used for their interesting optical properties, these observations show that an exhaustive structural and chemical analysis is compulsory in the aim to understand and optimize this system for the targeted applications.

## Figures and Tables

**Table 1:** Summary of all the synthesis parameters.

Sample	Y + Ln Amount					Solvent Volume				Heating			Type
	Y	Er	Yb	Gd		H_2_O	EtOH	OA					of
Name	(mole%)					(mL)				Time (h)	Temp (°C)		Container
NR00	50	2	18	30		17	20	20		2	200		75 mL Autoclave
NR01	80	2	18	0		17	20	20		20	200		75 mL Autoclave
NR02	80	2	18	0		17	20	20		2	200		75 mL Autoclave
NR03	75	2	18	5		17	20	20		2	200		75 mL Autoclave
NR04	75	2	18	5		17	20	20		20	200		75 mL Autoclave
NR05	65	2	18	15		45	50	50		2	200		200 mL Glass tube
NR06	65	2	18	15		45	50	50		2	200		200 mL Glass tube
NR07	65	2	18	15		45	50	50		20	200		200 mL Glass tube
NR08	50	2	18	30		45	50	50		2	200		200 mL Glass tube
NR09	50	2	18	30		4.5	5	5		2.5	20		20 Ml Glass tube
NR10	50	2	18	30		12	11	11		3	200		75 mL Autoclave
NR11	50	2	18	30		17	20	20		2	200		75 mL Autoclave
NR12	35	2	18	45		45	50	50		2	200		200 mL Glass tube
NR13	20	2	18	60		45	50	50		2	200		200 mL Glass tube

**Table 2:** Crystallographic data on synthesized phases of NaYF_4_ for phase identification and rare-earth (Re) ion site symmetry.

Phase	Space	Cell	Atomic		Occupancy	Re Site	COD	Ref.
	Group	Parameters (Å)	Positions			Symmetry	ID	
		a=b=	Y	2/3 1/3 1/4	0.75	$C_{3h}^{2}$		
Hexa-	P6_3_/m	5.99276	Na	2/3 1/3 1/4	0.25		1517675	[26]
gonal	176	c=	Na	0 0 z	0.25			
(β)		3.52281	F	x y 1/4	0.25			
		a=b=	Y	2/3 1/3 1/2	0.5	$C_{3h}^{2}$		
		5.99276	Y	0 0 0	0.75	$C_{3h}^{2}$		
Hexa-	P$\bar{6}$		Na	2/3 1/3 1/2	0.5		1517672	[26]
gonal	174		Na	1/3 2/3 z	0.5			
(β)		c=	F	x y 1/2	1			
		3.52281	F	x y 0	1			
		a=b=	Y	1/3 2/3 1/2	0.25	$C_{3h}^{1}$		
		5.9148	Y	0 0 0	1	$C_{3}^{1}$	1517674	[26]
Hexa-	P$\bar{6}$2m		Na	1/3 2/3 1/2	0.75			
gonal	189	c=	F	x 0 0	1			
(β)		3.52281	F	0 1/2	1			
		a=b=c=	Y	0 0 0	0.5	$O_{h}$		
cubic	Fm$\bar{3}$m	5.47000	Na	0 0 0	0.5		1517676	[27]
(α)	225		F	1/4 1/4 1/4	1			

**Table 3:** Expected and effective doping concentration of different NPs measured by EDX. Two lines in the tabular correspond to two population measured by EDX. The NPs sizes given come from the measured NPs and can differ from the mean values.

Sample	TEM Size (nm)		Aspect	Expected Doping (mol%)				Measured Doping (mol%)				Area in
Name	L	D	Ratio	Y	Er	Yb	Gd	Y	Er	Yb	Gd	Figure 3
NR00	300	50	6	50	2	18	30	40.2	9.4	18.2	32	
NR09	150	60	2.5	50	2	18	30	40.0	6.9	15.6	38.6	
NR11	200	30	6.6	50	2	18	30	41.5	8.4	17.5	32	1
	640	100	6.4					56	6.7	13.4	18	2
NR13	55	40	1.4	20	2	18	60	13.3	14.5	13.5	58	4
	130	35	3.7					23.9	14.6	13.9	47.6	3

**Table 4:** Main parameters of the studied NPs (with L for Length, D for Diameter, AR for Aspect Ratio) and the parameters from the Halder-Wagner graph with the apparent size and microstrain obtained for each of them. Here only the expected gadolinium concentration is given. (n.s. negative microstrain even if the value is very close to zero).

	TEM Size (nm)		AR	Gd	y=ax+b		Shape	Crystallite	Microstrain
	L	D		(mole %)	*a*	*b*	Factor	Size (nm)	
NR01	835	113	7.4	0	1.16	1.69	0.89	85	2.5 × 10^−4^
NR02	1390	234	5.9	0	0.92	0.42	0.82	108	1.3 × 10^−4^
NR03	718	108	6.6	5	1.35	0.29	0.77	74	1 × 10^−4^
NR04	935	160	5.8	5	0.98	0.46	0.83	102	1.35 × 10^−4^
NR05	375	70	5.4	15	1.69	0.79	0.81	59	1.7 × 10^−4^
NR06	465	70	6.6	15	1.44	2.9	0.76	69	3.4 × 10^−4^
NR07	640	100	6.4	15	1.3	0.8	0.81	77	1.8 × 10^−4^
NR08	400	55	7.2	30	2.01	0.36	0.8	50	1.2 × 10^−4^
NR09	156	62	2.5	30	2.01	−0.21	0.73	50	n.s.
NR10	130	70	1.9	30	2.04	0.53	0.77	49	1.5 × 10^−4^
NR11	260	55	4.7	30	2.21	1.08	0.80	45	2 × 10^−4^
NR12	137	45	3	45	2.83	0.72	0.77	35	1.7 × 10^−4^
NR13	60	45	1.3	60	3.9	−6	0.78	26	n.s.

**Table 5:** Main parameters of the studied NPs (with L for Length, D for Diameter, AR for Aspect Ratio) and the apparent size obtained by the anisotropic model. Here only the nominal gadolinium concentration is given.

	TEM Size (nm)		AR	Gd	Crystallite Size (nm)	
	L	D		(mol%)	min	max
NR01	835	113	7.4	0	77	100
NR02	1390	234	5.9	0	143	153
NR03	718	108	6.6	5	79	180
NR04	935	160	5.8	5	132	142
NR05	375	70	5.4	15	65	77
NR06	465	70	6.6	15	63	69
NR07	640	100	6.4	15	89	93
NR08	400	55	7.2	30	58	102
NR09	156	62	2.5	30	52	110
NR10	130	70	1.9	30	57	74
NR11	260	55	4.7	30	51	87
NR12	137	45	3	45	40	92
NR13	60	45	1.3	60	36	36
